# Network Dynamics of Attention During a Naturalistic Behavioral Paradigm

**DOI:** 10.3389/fnhum.2018.00182

**Published:** 2018-05-04

**Authors:** René Weber, Bradly Alicea, Richard Huskey, Klaus Mathiak

**Affiliations:** ^1^Media Neuroscience Lab, Department of Communication, University of California, Santa Barbara, Santa Barbara, CA, United States; ^2^Orthogonal Research and Teaching Laboratory, Champaign, IL, United States; ^3^Cognitive Communication Science Lab, School of Communication, The Ohio State University, Columbus, OH, United States; ^4^Department of Psychiatry, Psychotherapy and Psychosomatics, RWTH Aachen University, Aachen, Germany

**Keywords:** attentional networks, cognitive dynamics, network neuroscience, interactivity, video games, functional magnetic resonance imaging

## Abstract

This study investigates the dynamics of attention during continuous, naturalistic interactions in a video game. Specifically, the effect of repeated distraction on a continuous primary task is related to a functional model of network connectivity. We introduce the Non-linear Attentional Saturation Hypothesis (NASH), which predicts that effective connectivity within attentional networks increases non-linearly with decreasing distraction over time, and exhibits dampening at critical parameter values. Functional magnetic resonance imaging (fMRI) data collected using a naturalistic behavioral paradigm coupled with an interactive video game is used to test the hypothesis. As predicted, connectivity in pre-defined regions corresponding to attentional networks increases as distraction decreases. Moreover, the functional relationship between connectivity and distraction is convex, that is, network connectivity somewhat increases as distraction decreases during the continuous primary task, however, connectivity increases considerably as distraction falls below critical levels. This result characterizes the non-linear pattern of connectivity within attentional networks, particularly with respect to their dynamics during behavior. These results are also summarized in the form of a network structure analysis, which underscores the role of various nodes in regulating the global network state. In conclusion, we situate the implications of this research in the context of cognitive complexity and an emerging theory of flow during media exposure.

## Introduction

All visual and auditory stimuli are mediated in some way by attentional processing. Biologically, attention serves both as a general alertness mechanism and as a specific resource allocation mechanism (Raz and Buhle, 2006). These mechanisms serve two purposes relevant to real-time behavior. In a direct sense, they act to filter incoming perceptual information through the constraint of finite capacity (Lang, 2000). They also indirectly act to prioritize the order in which competing stimuli are processed. Observations of this self-organized cueing led (Petersen and Posner, 2012) to propose that the neural circuitry for attention forms a network with three distinct components: executive control, orienting, and alerting. While the activation of attentional networks in static stimulus paradigms has been carefully studied (Fan et al., 2005), simple demonstrations of how this activation is related to complex cognitive dynamics remain elusive.

We investigated the neural dynamics of network connectivity for attention while participants are engaged in a continuous activity while undergoing functional magnetic resonance imaging (fMRI). Participants played a first-person shooter video game (*Tactical Ops: Assault on Terror*; Villeurbanne, France) as primary task while a laser light presented to participants at randomized time intervals provided an exogenous distraction (secondary task). This task allowed us to examine the effect of ongoing distraction during a naturalistic continuous primary task on attentional network connectivity over time.

### Non-linear Attentional Saturation Hypothesis

**FIGURE 1:**
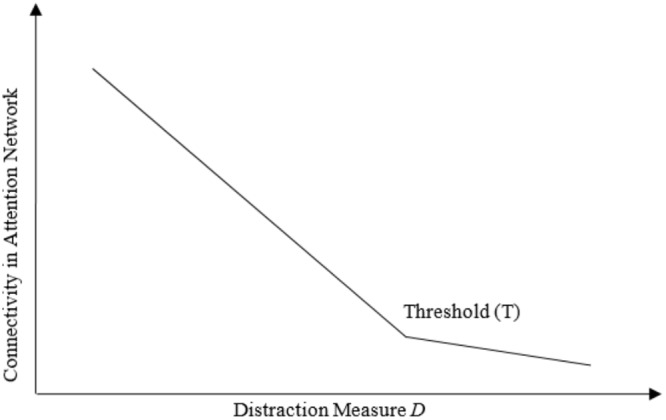
Relationship between Connectivity and Distraction. Hypothesized relationship between connectivity in attentional networks and distraction from a primary task.

Our experimental task can be broken down into two sub-components, each with unique properties. One stimulus (the video game) requires continuous attention, while the other stimulus (the laser light distractor) serves as a means to disrupt attention at random intervals. Within a limited capacity of attention framework (Lang, 2000), we can sketch two expected relationships between distraction and attentional capacity. To provide an intuition for our argument, let us consider that attentional capacity is characterized by a critical threshold value, beyond which attention changes significantly, and in turn affects connectivity between neural structures within attentional networks. With this framework in mind, we would expect that connectivity within attentional networks increases somewhat as distraction (*D*) decreases and nears a threshold value (*T*), but increases considerably as distraction (*D*) falls below threshold (*T*). Conversely, we would expect that increasing levels of distraction (*D*) actively force participants to split their attention between two or more tasks. Thus, a critical threshold value exists at which the magnitude of distraction exceeds capacity and causes a collapse of attentional function evident in the lack of connectivity between structures within attentional networks. We refer to this expectation as the Non-linear Attentional Saturation Hypothesis (NASH), formalized with a general linear statistical model and illustrated by the functional relationships between directed network connectivity and a distraction measure *D* shown in **Figure 1**. The assumptions that underpin the NASH are reliant upon the nature of attentional capacity and associated neuronal networks as defined in the literature. However, the NASH rests upon several methodological assumptions and conceptual definitions from the cognitive and network neuroscience literature to which we will now turn.

## Assumptions, Premises, and Definitions

### General Premises and Conceptual Background

In this manuscript, we focus on connectivity patterns within attentional networks (Mesulam, 2012). We define these connections in terms of effective connectivity, which accounts for the dynamic and task-modulated influence of one structure over another (Friston, 2011). While there are a number of potential interactions to explore (Spreng et al., 2013), this analysis will focus on defining the functional role of each region in the cognitive processing of potentially disruptive events (Meunier et al., 2009). We argue that over time (1) effective connectivity within attentional networks decreases (or increases) non-linearly with increasing (or decreasing) distraction, and (2) that effective connectivity exhibits a non-linear response at critical distraction values. This argument is based on two central premises.

The first premise is that cognitive functions are regulated by interconnected brain structures (Bassett and Gazzaniga, 2011). This study focuses on the importance of course-grained neural connectivity for examining attentional dynamics on a regional scale rather than a voxel-by-voxel basis (Power et al., 2011; Wig et al., 2011). In general, functional brain networks (FBNs, see Sporns et al., 2005; Friston, 2009; Plaza et al., 2014) involve multiple, distributed centers called nodes which play different roles in regulating cognitive functions and producing behaviors (Grobstein, 1988; Arbib et al., 1997). Based on evidence resulting from the study of default mode networks, structures within a functional network are likely to exhibit selective connectivity between regions and modules (Doucet et al., 2011). A study that treated attentional networks as having directed information flow (Yan and He, 2011), identified key hubs in attentional networks which included the inferior frontal gyrus, supplementary motor area, insula, and fusiform gyrus. More recently, a focus on right-lateralized attentional network connections has been implicated in maximizing the integration of information from disparate sources in the brain (Shine et al., 2016).

In general, FBNs require interaction between multiple distributed brain regions (Tononi et al., 1994). Such synchronous activity yields patterns that correlate with neuronal dynamics, particularly changes in brain state (Buonomano and Merzenich, 1998). Therefore, one of the key attributes of network organization is network connectivity (Buzsaáki, 2006). In the context of cognition, this means that strong connectivity might greatly strengthen the resiliency of attentional function with regard to distractions, while selective weakening or disruption of certain connections might lead to sudden shifts in function (for the general idea, see Van Essen et al., 1994; Betzel et al., 2016; for definition, see **Supplementary Presentation S1**).

In opposition to linear models of attentional capacity, the second premise argues that connectivity exhibits a robust response to distraction (laser light presentations) in the form of large distracter parameter values. One common feature of robust phenomena across various types of networks (for examples, see Holme and Kim, 2002) is a built-in response that keeps a system functioning even in the face of extreme disturbance or ambiguity. In the context of brain science, robustness is defined as the degree to which topological properties of a network can be reconfigured in response to perturbation (Bullmore and Sporns, 2012) or as a function of tolerance against errors (Albert et al., 2000; Achard et al., 2006). Recent results provide additional support for the view that robustness is a common feature of brain networks (Davison et al., 2015; Spielberg et al., 2015), and show that connectivity within the attentional network is dynamically reconfigured in response to cognitive processing (Spielberg et al., 2015; Telesford et al., 2016).

Attentional networks operate in a dynamic fashion, with functional regulation occurring on multiple time scales. This facilitates adaptation to new conditions, produces non-linear connectivity patterns between network structures, and allows brain networks that operate one way under normal conditions to acquire a new (or modified) functional state during disruption (Bilder and Knudsen, 2014). The ability of attentional networks to adapt over time may be optimized when the brain operates at a critical point between two modes of activity (Beggs, 2008; Haimovici et al., 2013; Gu et al., 2015; Cocchi et al., 2017). We predict that similar patterns occur in attentional networks. To test this, we use a laser light stimulus to introduce a distraction, which acts to force brain activity toward new states in a non-deterministic manner. Such an approach allows for the observation of specialized intra-network functions which emerge in specific contexts, such as how the insula mediates saliency during attentional switching (Menon and Uddin, 2010; Uddin, 2015), or how capacity limitations shift the locus of attentional control from posterior parietal cortex to frontoparietal cortex (Ptek, 2012).

### Empirical Assumptions

In terms of experimental design, we expect that the network components will shift from a linear response to a non-linear response with increasing amounts of distraction. Expertise in so-called “action” video games results from training, which has several augmentative effects on attentional capacity. These include rapid switching between tasks, enhanced acuity with respect to the visual field perimeter, increased speed of processing, and greater cognitive control (Green and Bavelier, 2015). By contrast, brain disorders such as ADHD (Sripada et al., 2014) or cognitive decline associated with aging and disease (Zuo et al., 2012) exhibit a lack of adaptive dynamics. In general, we can characterize adaptability in the brain as distributed connectivity characterized by smooth transitions between functional states in the face of fluctuating cognitive conditions (Parks and Madden, 2013; Davison et al., 2015).

In our study, we assume that continuous brain dynamics can be better understood using a continuous stimulus and a naturalistic experimental paradigm (Bartels and Zeki, 1998, 2004, 2005; Mathiak and Weber, 2006; Spiers and Maguire, 2007; Bohil et al., 2011; Maguire, 2012; Krakauer et al., 2017). This design provides a unique window into the functioning of brain activity unattainable using more traditional repeated-measures experimental paradigms with static stimuli and subtraction logic. Indeed, Hasson et al. (2009) have shown that collecting data using free-viewing conditions does not work against the dynamics and complexity of the brain, and provides a more realistic picture of neuronal activity and cognitive function. At the same time, we assume that a randomized presentation of a secondary distractor with button-press response during a naturalistic task is a trial presented in an event-related design (Mesulam, 1981; Friston et al., 1997; Dale, 1999; Hopfinger et al., 2004). To capture the dynamic nature of our dataset, we analyzed both the BOLD signal in each region of interest (ROI) and distraction across time. In this way, we observe the full effects of stimulus presentation and potential disruption of network connectivity. Methodological details on functional connectivity and the network structure analysis are described in **Supplementary Presentation S1**.

### Attentional Network Definition

For this study, temporally-specific information regarding a single attentive episode will be inferred from the attention models originally presented by Posner et al. (1987), Fan et al. (2002), and Petersen and Posner (2012). The model specifies three different types of attention that are sub-served by different functional networks; these include: alertness, orienting, and executive control. While the underlying neural correlates for these three functional subnetworks have been studied intensively (Fan et al., 2002), our experimental paradigm allows us to investigate how the effective connectivity patterns between structures within attentional networks during dynamic behavior. While the alerting and orienting networks are also interesting, activity in these networks may be driven both by the distractor stimulus as well as features of our video game stimulus (e.g., flashes on screen, gun shots). Instead, we are principally interested in the way capacity-limited attention is directed at either single or multiple stimuli. Accordingly, we focus on network connectivity patterns within the executive attention network, which sub-serves the top-down regulation of attentional resources in the pursuit of goal directed behavior.

## Hypotheses

### Statistical Hypotheses

To test for the hypothesized non-linearity in the relationship between distraction and connectivity, we use a non-linear, quadratic form of a general linear model to explain attentional functional connectivity. The NASH is expressed as three related statistical hypotheses related to Eq. (3). The first of these predicts that connectivity between pre-defined regions depends on the level of distraction as defined by the laser light stimulus:

H_1_: Connectivity depends on distraction, c ≠ 0

For the attentional network components unrelated to sensorimotor coordination, our general hypothesis not only predicts the existence of a dependency, but also specifically a reduction of connectivity. However, considering that the distractor task was a left-handed button-press task and involved sensorimotor coordination (sensing, planning, and execution of a button-press) we do not expect this reduction to occur in networks related to sensorimotor coordination (networks functionally connected to the cerebellum). We therefore expect that connectivity should decrease with increasing distraction for networks unrelated to sensorimotor coordination: c < 0 Analogously, we expect for networks that also contribute to sensorimotor coordination a positive relationship between connectivity and distraction, so that connectivity should increase with increasing distraction for networks related to sensorimotor coordination: c > 0.

Moreover, we can make predictions on non-linear behavior of the distraction-connectivity relationship. The NASH implies that increases in connectivity accelerate when distraction falls below a threshold *T* (see **Figure 1**). Thus, the relation has a non-linear term and the sign of the non-linear term is opposite to the sign of the linear trend. For attentional network components unrelated to sensorimotor coordination, we would expect:

H_2_: The curvature of an “increasing distraction-decreasing connectivity” relation is convex, i.e. if c < 0 then d > 0.

Correspondingly, for attentional network components related to sensorimotor coordination, i.e. for networks for which we expect an increase of connectivity with increasing distraction, we predict:

H_3_: The curvature of an “increasing distraction-increasing connectivity” relation is concave, i.e. if c > 0 then d < 0.

As we can see from H_2_ and H3, it is predicted that linear and non-linear coefficients will exhibit opposite signs, sign(*d*) = - sign(*c*). All hypotheses (H_1_, H_2_, and H_3_) were tested with second level statistics across the group. Calculations were performed in Matlab after a standard preprocessing pipeline for fMRI data (Weber et al., 2015b). Maximum levels of significance was set at *p* < 0.05 cluster corrected for multiple comparisons.

## Materials and Methods

### Study Detail

Different aspects of the data had been evaluated previously in (Mathiak et al., 2011). While the neuroimaging methods and sampling methodology are identical, in this study we perform new and additional analyses that go far beyond what was originally done in Mathiak et al. (2011). Specifically, we evaluate reaction time data (for the first time) as a method for characterizing level of distraction.

### Participants

Thirteen male volunteers (age 18–26, median 23) were recruited on the basis of previous experience with video games (15.1 ± 9.0 h/week) with ads posted at the local university and in video game stores. Inclusion criteria were: male, age between 18 and 26 years, playing at least 5 h weekly of video games, and right-handedness. Individuals with contraindication against MR investigations, acute or anamnesis of major neurological, psychiatric, or ophthalmologic disorders were excluded. All participants gave their written informed consent and the local ethics committee approved the study protocol. The study protocol was approved by the ethics committee of the University of Tübingen, Germany.

### Imaging Paradigm

The video game used in this experiment is a first person shooter simulation called *Tactical Ops: Assault on Terror* (Infogrames Europe, Villeurbanne, France). In this interactive simulation, participants play the role of a paramilitary operative armed with a machine gun. The objective is to rescue civilian hostages from their captors. The captors are armed with machine guns, and can shoot at the player. The focus is on realistic representation of the action. The game engine renders the action at 60 frames per second. The virtual environment provides visual stimuli that are of high fidelity, are highly-arousing, and require constant attention. Participants played *Tactical Ops* for five rounds, 12 min per round (60 min total), while in an MR scanner using a trackball device at their right hand to minimize motion artifacts due to hand-arm movements. While participants could freely choose how to play the game, each participant played the same game map with the same potential challenges. Given our selection of relatively high-skilled gamers (see above) who played identical game maps in similar ways, we argue that game difficulty/skill ratios did not vary much across all participants. Simultaneously, a secondary behavioral distraction task was performed (see below). Brain activity was measured by fMRI throughout game play and distraction. In addition, we recorded sound and video of the game play as well as response times in the distraction task and synchronized all data with the fMRI trigger signal.

### Behavioral Distractor Task

As subjects interacted with the continuous primary stimulus, a red laser projected a light point into the periphery of the visual field (right upper quadrant) at random time intervals until participants responded. Delays in laser light presentations followed a Poisson distribution with an average time delay of 10 s (I_p_) after the last button press. The Poisson distribution was chosen to ensure equal probabilities for laser light presentations at any moment. The time intervals were independent from changes in the primary task, but required the subject to respond in the fastest possible time by pressing a button with the left hand. Pressing the response button reset the timer on the laser light and initiated another trial. As the secondary distractor task was performed inside the MR scanner during ongoing game play, it served as an incongruent stimulus relative to the main action in the simulation. The action in the video game itself is the primary task, and required regulation by the executive attention network. The mean time interval between laser light presentations (I_p_) and the mean response time to each presentation of the laser light (I_r_) was used to calculate the distraction parameter *D*.

### Definition of Distraction

The distraction parameter (*D)* used to model fMRI data is determined by a response time measure in a secondary distractor task. *D* is defined as a multiplicative distraction index calculated over a constant 10-s sliding (or overlapping) window (Δt). The index calculates the inverse of the mean time interval between laser light presentations (*I*_p_) multiplied by the mean response time to each presentation of the laser light (*I*_r_):

(1) $$\begin{array}{c} D_{\Delta t}=\frac{1}{I_{p}\times I_{r}} \end{array}$$

As such, distraction is defined as the various demands on attentional capacity throughout the course of the task. In other words, distraction is measured as the inverse of the time between events multiplied by the time needed for a response. The more laser light presentations (*I_p_* → 0) and the faster participants’ response to those laser light presentations (*I_r_* → 0) the higher the distraction from the primary experimental task and the higher is *D* in sliding window Δt. Characterizing distraction in this way allowed us to define a continuous measure of distraction that spanned the entire experimental paradigm. This approach is consistent with classic computational modeling approaches to fMRI data where brain data are modeled using a continuous regressor.

### fMRI Data Acquisition

For this study, fMRI was conducted at a magnetic field strength of 3 Tesla (Magnetom TRIO, Siemens, Erlangen, Germany). Multi-echo single-shot echo-planar imaging (EPI; echo times = 23, 40, and 62 ms) with dynamic distortion correction (Weiskopf et al., 2003) and dephasing compensation (Mathiak et al., 2004) reduced artifacts and increased sensitivity. Whole brain coverage with 24 interleaved slices (repetition time = 2.25 s) and spatio-temporal oversampling reconstruction resulted in an apparent time resolution of 1.13 s after spatial filtering. For reference, we acquired anatomical data of each participant before the functional sessions (T1-weigthed 3D-MPRAGE, 256 × 224 × 160 matrix with 1 mm isotropic voxels).

#### ROI Analysis

The ROI analyses that are reported here rely on *a priori* assumptions about the brain areas and networks involved. As discussed above, we concentrate on the executive attention network model proposed by Posner et al. (1987), and thus rely on functions and localizations suggested in Fan et al. (2002, 2005). The attention-distractor task in our paradigm is conducted with the left hand since visuospatial attention is right lateralized (Thiebaut de Schotten et al., 2011). For this reason, our attentional network is biased toward executive attention components in the right hemisphere. The ROIs have been localized according to the suggested anatomical localization in Collins (1994), and are represented as standardized Montreal Neurological Institute (MNI) coordinates: [22, -27, 3] mm for the thalamus (Thal); [16, 4, 44] mm for superior frontal gyrus (SFG); [36, 26, 15] mm for superior parts (IFGs) and [34, 20, 5] mm for inferior parts of the inferior frontal gyrus (IFGi); [44, -58, 1] mm for lateral parts (FFGl) and [36, -60, 1] mm for medial parts of the fusiform gyrus (FFGm); [0, -62, -32] mm for the cerebellum (Cere); [36, -5, 50] mm for middle frontal gyrus (MFG); and [6, 36, 26] mm for anterior cingulate cortex (ACC). In order to compare the intensity of activation over time, time-series data from nearest activation maxima in the normalized and smoothed functional images were extracted. While the restriction to the right hemisphere corresponds to previous research in this area, and reduced confounds of inter-hemispheric connections (Rosen et al., 1999), we also extracted all corresponding ROI’s in the left hemisphere. This allowed us to test our models for consistency in both hemispheres.

### Definition of Connectivity

In the simplest of terms, connectivity can be understood as the correlation (or statistical dependency) between two neural time-series. This is known as functional connectivity (Friston, 2011). Studies using functional connectivity and naturalistic stimuli have demonstrated that BOLD signal correlations are likely to yield course-grained temporal information about the information transfer between brain regions (Bartels and Zeki, 2004). However, a recent push in the neuroimaging literature has been to more completely characterize connectivity patterns within networks. Effective connectivity (Friston, 2011) analyses account for the dynamic and task-modulated influence of one network structure over another. While a number of methods exist for testing effective connectivity, we rely on a general linear model logic where the task-dependent neural time-series of a given ROI is correlated with other neural time-series (Friston et al., 1997).

### Brain Connectivity as Psycho-Physiological Interactions

We use a model of Psycho-Physiological Interactions (PPI) to characterize effective connectivity. The PPI model (O’Reilly et al., 2012) can be generalized in the following form (Eq. 2):

(2) $$\begin{array}{c} Y=(P_{\mathrm{sy}})\beta_{1}+(P_{\mathrm{hy}})\beta_{2}+(P_{\mathrm{sy}})*(P_{\mathrm{hy}})\beta_{3}+e \end{array}$$

where β_1_ represents the parameter estimate for the psychological variable main effect, β_2_ is the parameter estimate for the physiological variable main effect, β_3_ represents a parameter estimate for the interaction term, *P*_sy_ is the psychological variable of interest (e.g., attention), and *P*_hy_ is the physiological variable of interest (e.g., neural time series within a given ROI), and *Y* is the combined psychological and physiological effect from one ROI on another ROI.

### General Linear Model

For our analysis we choose ROIs *a priori* for the executive attention network as suggested in Fan et al. (2002, 2005). This topographically conservative approach minimized the chance of false positives and allowed for the interpretation of the connectivity analysis within the framework of established network models. In line with psycho-physiological interaction model of connectivity (Friston et al., 1997, 1998; O’Reilly et al., 2012), we consider a regression model that links the BOLD signal in the target region (ROI_T_) with that of the source region (ROI_S_), the distractor parameter *D*, and their interaction.

The General Linear Model in our analysis is similar to the general PPI model (Eq. 3):

(3) $$\begin{array}{c} {\mathrm{ROI}}_{T}=a*{\mathrm{ROI}}_{S}+b*D+\;(c*D+\;d*D^{2})*{\mathrm{ROI}}_{S}+\varepsilon \end{array}$$

Since this is a non-linear equation, we assume theicients from the model were used as a measure of connectivity between brain ROIs.

### Public Access of Data

The data featured in this study is publically available in the Open Science Framework (OSF) in the form of two CSV files containing all extracted ROIs and our distraction measures (one file for each hemisphere). This data has been used to estimate the model parameters reported in this article. The URL of this repository is https://osf.io/435kr/. The digital identifiers are doi: 10.17605/OSF.IO/435K and ARK: c7605/osf.io/435kr.

## Results

### Manipulation Check

The success of our study rests on participants treating the video game as a primary task, and the distraction measure as a secondary task (Lang, 2000). Subjects were asked a series of 9-point (1 = totally disagree, 9 = totally agree) self-report measures to determine the extent to which they were drawn into the game. These data generally support the conclusion that subjects treated the video game as a primary task. Specifically, subjects were asked to evaluate the extent to which the study was fun (*M* = 7.4, *SD* = 1.6), the study was interesting (*M* = 7.9, *SD* = 1.4), and that they would participate in a similar study again (*M* = 7.6, *SD* = 2.1). Measures of involvement with the video game were also above scale mean in that participants: felt like they were acting in the environment rather than controlling a game (*M* = 4.7, *SD* = 2.4), felt present in the game environment (*M* = 5.7, *SD* = 2.3), felt like they were not aware of the real environment (*M* = 5.6, *SD* = 3.5), and that they felt like the game required all of their attention (*M* = 5.6, *SD* = 2.3). Taken together, these self-report data suggest a successful manipulation in that subjects treated the video game as a primary task, and our distraction measure as a secondary task.

### Distraction Measure

**FIGURE 2:**
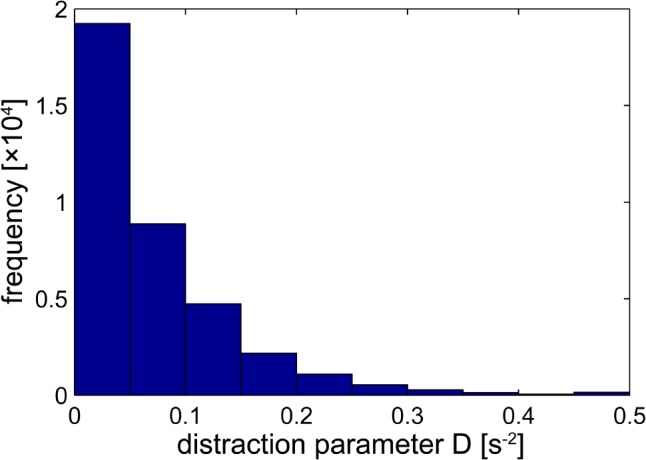
Distribution of the distractor parameter *D*. For each volume in the functional magnetic resonance imaging (fMRI) time series *D* is calculated (using a 10-s sliding window) as the inverse of the product of response time and time between distractor presentations. 95% of the values fall below 0.2128.

The behavioral distractor task was a light point projected by a red laser requiring a speeded button-press response of the left hand. The mean response time (*I*_r_) was 1158.3 ms (90% interval, 5th to 95th percentile: [434.7, 16739.6]) with slower responses after longer Inter-Stimulus Intervals (ISIs – *r* = 0.55, *p* x < 0.0001). The derived distraction measure *D* was on average 74.4 ms^-2^ (90% interval, 5th to 95th percentile: [2.2, 212.8] ms^-2^). A 90% interval for the *I*_r_ and derived distraction measures provides a better insight into the hyperbolic nature of its distribution with respect to response times within and between participants. **Figure 2** shows the distribution of the distraction parameter *D* across our participants.

### Brain Imaging Data

#### Changes in Connectivity

**FIGURE 3:**
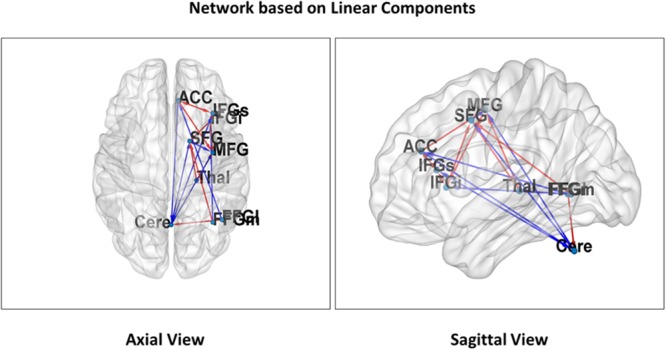
Linear attentional networks. Nine regions of interest representing *linear relationships* in the executive centers of our attentional network are projected on both an axial **(Left)** and sagittal **(Right)** slice of the MNI reference (THAL, thalamus; SFG, superior frontal gyrus; IFGs, superior parts of the inferior frontal gyrus; IFGi, inferior parts of the inferior frontal gyrus; FFGl, lateral parts of the fusiform gyrus; FFGm, medial parts of the fusiform gyrus; CERE, Cerebellum; MFG, middle frontal gyrus; ACC, anterior cingulate cortex). The arrows visualize connections that showed a significant *linear* effect of the distraction parameter. Red arrows indicate a decrease and blue arrow an increase of connectivity with higher distraction. The hypothesized reduction of connectivity with increasing distraction is mainly found in frontal network areas whereas cerebellar connections show an increase. The latter finding may be due to increased motor-coordination as a result of increased stimulus/distraction-response events. In order to compare the intensity of activation over time, time-series data from nearest activation maxima in the normalized and smoothed functional images were extracted.

**FIGURE 4:**
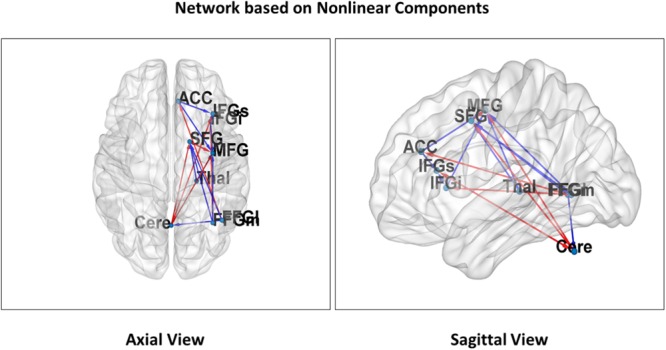
Non-linear attentional networks. Nine regions of interest representing *non-linear relationships* in the attentional network are projected on both an axial **(Left)** and sagittal **(Right)** slice of the MNI reference. The arrows represent connections that showed a significant *non-linear* effect of the distraction parameter. Red arrows indicate a concave and blue arrow a convex relationship of connectivity with distraction. All connections are significant for both linear and non-linear coefficients and showed the opposite sign (see **Figure 3** for labels).

**Figure 3** illustrates significant linear changes of connectivity in attentional network components in the right hemisphere as a result of varying distraction levels over time (coefficient *c* in the general linear model). **Figure 4** illustrates significant non-linear changes of connectivity in attentional network components in the right hemisphere as a result of varying distraction levels over time (coefficient *d* in the general linear model).

In Hypothesis 1 (H_1_) we predicted reduced connectivity with increasing distraction for attentional network components unrelated to sensorimotor coordination. This pattern did indeed emerge for most local network connections, particularly between frontal areas and thalamo-frontal projections (red arrows in **Figures 3, 4**). In contrast, increased connectivity emerged mainly in the cerebellar-cortical connections reflecting motor-coordination networks (blue arrows in **Figures 3, 4**). **Table 1** shows the coefficients of all models and their significance at the group level. Taken together, these findings support H_1_ (unrelated to sensorimotor coordination for local connections and related to sensorimotor networks for long-range connections).

**Table 1:** Group means of linear connectivity coefficients (*c* coefficients).

*c*	Thal	SFG	IFGs	FFGm	IFGi	Cere	MFG	FFGl	ACC
Thal		–1.14**	–0.33	0.01	–0.05	–0.57	–2.34**	0.59	–0.20
SFG	–0.51		–0.26	0.13	–0.38	0.50	1.14*	–0.95	–0.22
IFGs	–0.55	–0.06		–0.28	–0.64**	1.03***	–0.38	0.73	–0.41*
FFGm	0.31	–0.81**	–0.38		0.83*	–0.35	–1.65	1.11	–0.28
IFGi	0.06	–0.89*	–1.38***	0.37		0.55	–2.51*	0.97	–0.33
Cere	–0.26	1.04***	1.79***	–0.22	0.67		2.41***	–1.1	0.41
MFG	–0.09	0.24	0.22	–0.01	–0.22	0.23		–0.38	–0.06
FFGl	0.24	–0.51	0.66	0.02	0.49	–0.71*	–0.40		0.64**
ACC	0.03	–0.73	–1.66**	–0.12	–0.13	0.67*	–2.20**	1.67	

*Columns are predicted by rows: for example, SFG ∼ (c^∗^D + d^∗^D^*2*^)^∗^Thal = *(-1.14^∗^D + 2.64^∗^D^*2*^)^∗^Thal (for d coefficient, see **Table 2**). Hypothesis H_*2*_: sign(d)* = *-sign(c) is supported for all significant models. THAL, thalamus; SFG, superior frontal gyrus; IFGs, superior parts of the inferior frontal gyrus; IFGi, inferior parts of the inferior frontal gyrus; FFGl, lateral parts of the fusiform gyrus; FFGm, medial parts of the fusiform gyrus; CERE, cerebellum; MFG, middle frontal gyrus; ACC, anterior cingulate cortex*. ^∗^*p* < 0.05, ^∗∗^*p* < 0.01, ^∗∗∗^*p* < 0.001.*

**FIGURE 5:**
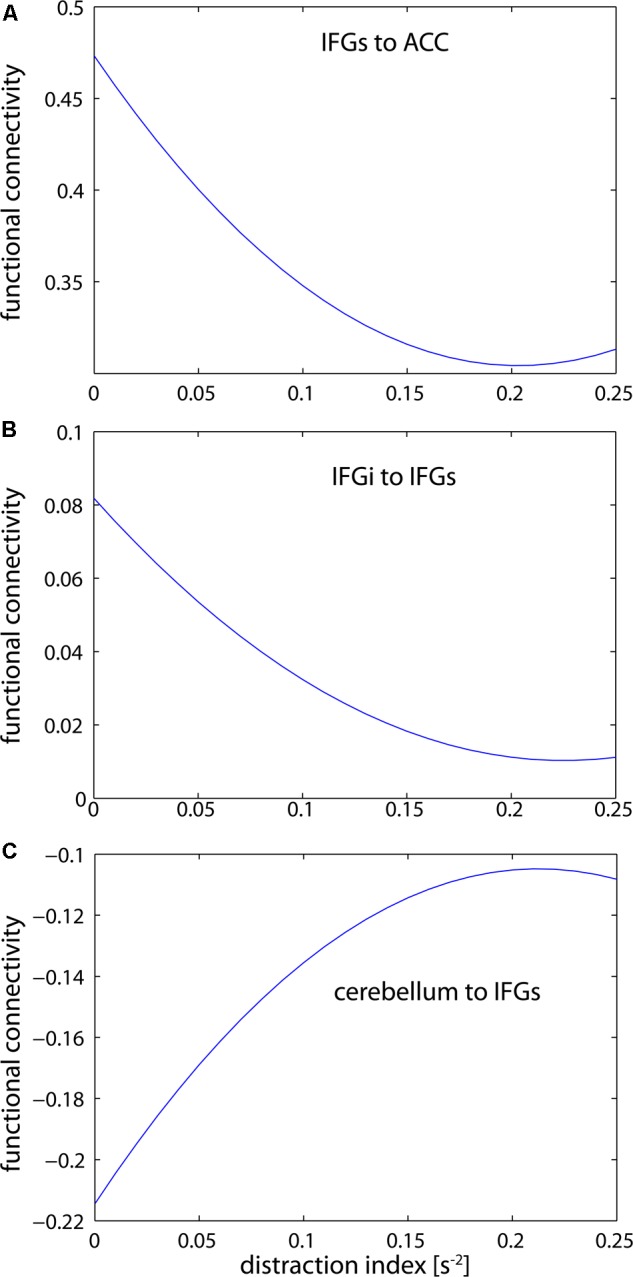
Estimations of the distraction-connectivity function. Inter-frontal connectivity falls off but levels out with increasing distraction **(A,B)**. In contrast, cerebellar-frontal connectivity increases but also reaches a maximum level **(C)**. Interestingly, the connectivity estimate is negative in the latter path; thus the absolute correlation is also reduced with increasing distraction. (IFGs, superior parts of the inferior frontal gyrus; IFGi, inferior parts of the inferior frontal gyrus; CERE, cerebellum; ACC, anterior cingulate cortex).

We confirm that H_2_ predicts that the curvature of a convex “increasing distraction-decreasing connectivity” relation (**Figures 5A,B**). We also confirm that H_3_ predicts that the curvature of an “increasing distraction-increasing connectivity” relation is concave (**Figure 5C**). The consequences of these findings can also be observed in **Figure 2**. As the value of *D* tends toward 0.5 in **Figure 2**, the strength of connectivity between structures tends toward 0.

We also tested our results for consistency in the left hemisphere. The analysis replicated the right-hemispheric pattern with generally reduced effect sizes. Both the linear (*r* = 0.58, *p* < 0.001) and the quadratic coefficient estimates (*r* = 0.53, *p* < 0.001) were highly correlated between the left and the right hemisphere.

### Organization of Attentional Networks

Another way to further quantify network connectivity for the significant linear and non-linear connections is shown in **Figures 3, 4**, respectively. The network structure analysis involves a rank-order evaluation of significant coefficients as defined in **Tables 1, 2**. For each set of coefficients (linear and non-linear network connectivity), a series of hyperbolic regressions were conducted. Each ROI (node) is characterized by summing all inbound and outbound edges (connections) converging upon that location in the attentional network (**Table 3**). The resulting vector is then ordered from largest to smallest without regard for node identity. We expect these network topology profiles to deviate from randomized sets of inputs and outputs in the following manner: randomized connections should yield a *R*^2^-value close to 0, while hierarchically-structured sets of connection should yield a *R*^2^-value approaching 1.

**Table 2:** Group means of non-linear connectivity coefficients (*d* coefficients).

*d*	RThal	RSFG	RIFGs	RFFGm	RIFGi	RCere	RMFG	RFFGl	RACC
RThal		2.64**	0.62	0.62	0.62	2.11	7.61**	0.61	0.73
RSFG	0.41		–0.89	–1.38	0.19	–3.51	–5.50*	2.89	0.37
RIFGs	0.50	–0.36		–0.21	1.41*	–2.43**	5.17	–2.61	0.54
RFFGm	–1.14	2.60*	0.17		–2.05*	3.86	5.21*	–2.25	0.33
RIFGi	–0.54	2.96	4.81***	0.07		1.33	11.59*	–3.47	0.66
RCere	–0.05	–3.53***	–5.57***	1.32	–2.61		–10.30***	1.94	–0.93
RMFG	–0.29	–0.20	–0.12	–0.24	0.94	–1.57		0.60	0.27
RFFGl	–1.14	2.52**	–1.99	–0.65	–3.30	2.61*	1.79		–3.04**
RACC	0.54	2.26	4.09***	0.37	0.48	1.43	11.57**	–4.66	

*Columns are predicted by rows: for example, SFG ∼ (c^∗^D + d^∗^D^*2*^)^∗^Thal = *(-1.14^∗^D +2.64^∗^D^*2*^)^∗^Thal (for c coefficient, see **Table 1**). Hypothesis H_*2*_: sign(d)* = *-sign(c) is supported for all significant models*. ^∗^*p* < 0.05, ^∗∗^*p* < 0.01, ^∗∗∗^*p* < 0.001.*

**Table 3:** Results of the network structure analysis.

Linear				Non-linear			
	Inbound	Outbound	Total		Inbound	Outbound	Total
Cere	3	3	6	RCere	2	3	5
FFGl	0	2	2	RFFGl	0	3	3
FFGm	0	2	2	RFFGm	0	3	3
MFG	5	0	5	RMFG	6	0	6
Thal	0	2	2	RThal	0	2	2
SFG	4	1	5	RSFG	4	1	5
ACC	2	3	5	RACC	1	2	3
IFGs	3	2	5	RIFGs	3	2	5
IFGi	2	3	5	RIFGi	2	2	4
Edges	–	–	19	Edges	–	–	18
*R^2^*	0.74	0.42	0.72	R^2^	0.87	0.70	0.88
*m*	–	–	3	*m*	–	–	4
$\bar{C}$	–	–	0.21	$\bar{C}$	–	–	0.23

*Network structure analysis outcomes based on linear (significant c-coefficient) and non-linear (significant d-coefficient) network connectivities. Number of edges: sum of totals for each network type divided by 2. “Inbound” and “Outbound”: direct of connections relative to each node. Detailed descriptions for m (network diameter), $\bar{C}$ (average clustering coefficient), and R^*2*^ are located in **Supplementary Presentation S1***.

For the directed linear graph (shown in **Figure 3**), a hyperbolic regression (**Table 3**) for all connections fits the data with an *R*^2^-value of 0.72 (*p* < 0.001). For just the inbound nodes, the *R*^2^-value is 0.74 (*p* < 0.001). For just the outbound nodes, the *R*^2^-value is 0.42 (*p* < 0.001). This suggests on average, inbound connections tend to converge on selected hubs, while their sources come from throughout the network. A hyperbolic regression analysis (**Table 3**) for all connections in the directed non-linear graph (shown in **Figure 4**) yields an *R*^2^-value of 0.88 (*p* < 0.001). The *R*^2^-value for inbound nodes only is 0.87 (*p* < 0.001). The *R*^2^-value for outbound nodes only is 0.70 (*p* < 0.001). This suggests that the non-linear version of the network contains more influential hubs (greater centralized control), each with a more selective set of sources. The data in **Table 3** also reveal this hierarchical structure through a comparison of increased network diameter (*d*) between the directed linear graph (*d* = 3) and directed non-linear graph (*d* = 4).

### Non-linearity in Attentional Network Connectivity

A comparison of linear components (**Table 1** and **Figure 3**) and non-linear components (**Table 2** and **Figure 4**) resulting from our GLM results in a similar pattern of network connectivity. However, the differences that do exist between the *c* and *d* parameters demonstrate significant non-linear changes of connectivity in our attentional networks that are due to varying distraction levels over time.

The local frontal and thalamo-frontal connections which showed a decrease in connectivity (*c* < 0) revealed a significant convex relationship (*d* > 0) while the long-range projections for motor coordination (with linear increase; *c* > 0) were governed by a concave relationship (*d* < 0) between connectivity and levels of distraction (see **Tables 1, 2**).

Importantly, all commonly emerging pathways showed opposite signs for linear increase versus curvature. In other words, the increase or decrease of connectivity due to distraction was limited by the non-linear term, and was thus dependent on the level of distraction. The independent graphs in **Figure 5** illustrate this relationship for three selected pathways. As second order approximations of the NASH, **Figures 5A,B** (IFGs–ACC and IFGi–IFGs) support Hypotheses 2 (H_2_), while **Figure 5C** (Cerebellum–IFGs) support Hypothesis 3 (H_3_).

## Discussion

In this study we have presented both a rationale and technique that permits us to investigate the dynamics of attention in a complex, immersive environment (a video game) by mapping psychophysiological responses to an attentional network (Friston and Buchel, 2000). This allows us to investigate both regional and global responses to sustained attention and selective disruption in a statistically rigorous manner. For both right- and left-hemispheric executive attentional networks, we find support for NASH. Local frontal network connectivity during a continuous experimental task (playing of a first-person video game) decreases with increasing distraction in a simple incongruent stimulus-distraction task. In contrast, cerebellar projections subserving motor-coordination show increased connectivity with increasing distraction. Overall, the result for attentional and sensorimotor networks shows a similar but inverse relationship.

### Results in the Context of NASH

As predicted by the NASH, the relationship between network connectivity and distraction is non-linear and convex. While we concede that the primary experimental task (playing a video game) might also be activating alerting and spatial orientation networks to some degree, one should keep in mind that first-person video games are designed to fully capture alertness and orientation in any moment (especially when played in an experimental setting under continuous observation). This means that either there is insufficient variation of activity in attentional networks given the nature of our primary task, or that even a slight distraction from the primary task might lead to complete disruption within alerting and orienting networks.

Other secondary distractor tasks, such as asking participants to execute simple repetitive actions simultaneously to the primary task, might be more suitable for studying those networks (Weber et al., 2015a). The observed negative and increasing contribution of the cerebellum with increasing levels of distraction may be due to the strong inhibitory function of that brain structure (Montarolo et al., 1982). This would explain why an increase in connectivity under increasing distraction levels was expressed as a lower negative correlation (see **Figure 5C**). Moreover, this difference in connectivity patterns between short and long-range structures provides further clarity to the emerging literature attempting to characterize the local and global network characteristics of attentional systems (Hermundstad et al., 2014; Davison et al., 2015). Similarly, previous work has shown that the video game used in this study yields high levels of motivation (Klasen et al., 2012). If true, then our results also fit within the emerging perspective that hierarchical and reciprocal network dynamics within the frontal cortex subserve motivated behavior (Kouneiher et al., 2009).

The convex and concave relationships in **Figures 3** (H_2_), **4** (H_3_), respectively, provide a dynamic view of how distraction can affect attentional networks. We can also understand the effect of distraction on attentional processing in neuropsychological terms. In particular, distraction tends to play a much more complex modulatory role with respect to the attentional network. While previous studies have not accounted for the effects of varying degrees of distraction on attention, the broader mechanisms have been identified. In studies of pain perception (Bantick et al., 2002), it has been found that distraction can mitigate pain. This is typically understood as an attentional modulation mechanism (Torta et al., 2017) consistent with the load theory of attention (Lavie, 2010). The load theory of attention suggests a distinct role for distractor processing as the preservation of normal cognitive functioning in the face of distraction, which is itself impacted by relative amounts of cognitive load (Lavie, 2005).

When distractor processing has an effect on attentional processing, the effects are heterogeneous with respect to various parts of the attentional network. For example, high cognitive load experienced in the frontal regions of the attentional network can increase distractor processing, while high amounts of cognitive load in other regions can decrease distractor processing (Van den Heuvel and Sporns, 2011). In context, H_2_ means that a breakdown of attention is equivalent to increased behavioral distraction. While this relationship is linear for normal levels of distraction, H_3_ predicts that a robustness mechanism may also contribute to limited attentional resources for very high levels of distraction. Thus, **Figure 2** shows that activity amongst the network nodes is retained for distraction parameter values between 0.2128 and 0.5 (beyond the 95th percentile).

Given this context, we can say that **Figure 2** suggests that long-tail connectivity achieved at greater values of the distraction parameter is restricted to certain parts of the network. For those connections (**Figures 5A,B**) that are consistent with H_2_ (increasing distraction, decreasing connectivity), the ROIs demonstrate an ability to work independently. These centers tend to be in the frontal areas of the brain, which is consistent with the notion of distraction processing. Connections between ROIs consistent with H_3_ (increasing distraction, increasing connectivity) involve centers that require interdependence as cognitive processing is assisted through offloading. While network statistics suggest that this effect is small, changes in the demands of cognitive processing result in regions with a greater number of connections in the linear case becoming slightly more connected. Meanwhile, regions with fewer connections in the non-linear case become relatively less connected with an emphasis on retaining outbound (directed) connections (Kelso and Engstrom, 2006). From a systems-level perspective, network stability is buffered by emphasizing more connected parts of the network and de-emphasizing peripheral parts of the network.

### Revisiting the Premises of NASH

Having found support for NASH, it is worth recalling that the hypothesis is based on two central premises. The first premise suggested that cognitive functions are regulated by connected brain structures. Since we have shown that connectivity in attentional networks decreases non-linearly for a certain range of increasing *D* values, something keeps network connectivity from collapsing entirely during this response phase. Our graphs in **Figure 5** provide a snapshot of three effects on the attentional network: intra-regional connectivity (within the inferior frontal gyrus), inter-regional connectivity for a non-hub region of the attentional network topology (between the inferior temporal gyrus and anterior cingulate cortex), and inter-regional connectivity representing the sensorimotor cortex activity. The aforementioned intra-regional connectivity always results in very low values, while inter-regional connectivity for both the attentional and sensorimotor networks becomes dampened to a greater extent. Overall, the trend shows a dampening with regard to rare, high-magnitude distracter events that occur at a similar distracter parameter value. This suggests an inherent response mechanism that emerges from the dynamic, complex nature of the brain (Rasche and Gegenfurter, 2010).

The second premise suggested that the relationship between distraction and attentional network connectivity exhibits a non-linearity that demonstrates a robust response at a critical threshold value. Our combination of naturalistic behavior, short repetition time, and presence of noise in the form of our distractor task (for use in perceptual systems, see Aertsen et al., 1989) allows us to observe a dynamic cognitive response influenced by systematic noise. Neuronal noise can play a role in selectively modifying connectivity patterns (Chialvo et al., 2008; Faisal et al., 2008), or even drive transitions between two network states (Raz et al., 2005; Chialvo, 2010). One source of these noise-driven non-linearities is the top-down regulation of attention. For example, the selective top-down role of hypnotic stimuli [see also (Kosslyn et al., 2000; Raz and Buhle, 2006) suggests that centralized top-down control of attentional networks during periods of heavy information processing such as in multitasking] are triggered by reaching critical threshold values of distraction rather than purely incremental distractions by a secondary task (Kelso and Engstrom, 2006). Such saturation effects have already been observed for automatic cognitive processes (Mathiak et al., 2005). Exactly how and when we encounter the critical threshold can only be determined in context, but the general mechanism implies non-linearity. Likewise, exogenous cues similar to our distractor task have been found to augment and improve pre-attentive function over time in recognition tasks and video-game expertise (Brawn and Snowden, 1996; Green and Bavelier, 2003). In this case, robustness may result from a feedback-dependent selective mechanism within and between brain regions (Edelman, 1987; Sporns et al., 2000).

### Broader Implications

There is still much to learn about brain dynamics and complex cognition in real-world environments (Spiers and Maguire, 2007). For instance, how do naturalistic behaviors relate to dynamic brain activity and known principles of connectivity? From a systems-level perspective, work on the concept of highly-optimized tolerance (Carlson and Doyle, 1999) suggests that complex systems that produce power-law responses often yield three characteristic traits: a high level of efficiency and robustness, hypersensitivity to unanticipated disruptions, and specialized topological configurations. These specialized network structures arise as a response to disruptions occurring at multiple temporal scales simultaneously (Doyle and Csete, 2005). In instances as diverse as aging, ADHD, and disease, FBNs tend to become less interconnected and more centralized, where peripheral regions are mediated by the activity of hub regions (Parks and Madden, 2013). In general, adaptability in the brain is characterized by distributed connectivity characterized by smooth transitions between functional states in the face of fluctuating cognitive conditions (Bartels and Zeki, 1998; Spielberg et al., 2015).

The approach presented here contributes to this inquiry by simulating real-world behaviors in an interactive virtual environment and developing advanced metrics for the analysis of cognitive dynamics. This research can also inform emerging communication and media theories, which at their core are dependent upon advances in understanding attentional network dynamics. As media such as video games and virtual reality become increasingly immersive, ubiquitous, and continually stimulative, we require an understanding of attentional networks at the level of first-principles. The Synchronization Theory of Flow (Weber et al., 2009) may serve as a recent example. As currently understood, flow (Csikszentmihalyi, 1990) is a rewarding motivational state requiring high levels of attention, reward processing, and cognitive control (goal planning, goal maintenance, performance monitoring, response inhibition). Synchronization Theory argues that “flow” emerges from the spontaneous emergence of a global cognitive state. We have demonstrated in this paper that synchronization in attentional networks, driven by connectivity, indeed occurs at a critical level of distraction. Similar non-linear dynamics can also be found between motivation and task-related attentional engagement (Lang, 2000; Lang et al., 2006). For example, motivational stimuli have been shown to exert influences on neural processing (Collins, 1994; Mathiak et al., 2013; Ulrich et al., 2013; Yoshida et al., 2014). However, the extent to which motivation modulates the strength of network connectivity within reward and cognitive control networks and drives shifts between networked brain states optimized for task engagement or disengagement is currently unknown. Aside from being useful in fields ranging from anthropology to human-computer interaction, the type of naturalistic neurobehavioral quantification introduced in this paper may also be particularly well suited for addressing these types of questions for examples, see (Krakauer et al., 2017).

## Author Contributions

RW and KM: experimental design and execution. RW, BA, and KM: concept and analysis. RW, BA, and RH: manuscript writing. RW and RH: figures and open dataset. RW, BA, RH, and KM: methods and supplemental materials.

## Conflict of Interest Statement

The authors declare that the research was conducted in the absence of any commercial or financial relationships that could be construed as a potential conflict of interest.

## Supplementary Material

PRESENTATION S1 Supporting Methods. Methodological detail, including mathematical and descriptive details for measures and statistical models (PDF File).
