# Supplementary material for: Network Dynamics of Attention During a Naturalistic Behavioral Paradigm
Source: Front Hum Neurosci. 2018 May 4;12:182. doi: 10.3389/fnhum.2018.00182 (PMC5946671; doi:10.3389/fnhum.2018.00182)
Supplement: PRESENTATION S1 — Supporting Methods. Methodological detail, including mathematical and descriptive details for measures and statistical models (PDF File). [file Presentation_1.pdf]

PRESENTATION 1

Definitions and Measures

Definition of Disruption

Disruption (R) can be defined mathematically as a conditional threshold

$$R = \begin{cases} if\ D_{\Delta t} > T, then\ R = 1 \\ else\ I_p, I_r \to 0 \end{cases} \tag{1}$$

where T is a threshold level that is *a priori* undefined.

We rely on a conceptual application in this paper, which assumes that disruption is the (intentional or unintentional) inability to divide attention between experimental and distractor task in a given time interval (Δt) and is a function of distraction levels exceeding a threshold level T. No disruption occurs when there is limited distraction. Participants are able to divide their attention between the distractor task and interacting with the virtual environment in a sufficient manner. Such disruptions are expected when a participant either cannot cue tasks appropriately or decide which action to perform next.

Network Structure Analysis

A quantitative analysis of network structure is conducted on the results of the linear model. Several network statistics are calculated from the statistically significant interactions between attentional network nodes as identified by the linear and nonlinear models (considered separately). Network structure is then analyzed in two ways: the first by examining the rank-ordered number of connections (inputs to each node, outputs from each node, and their sum for each node) for each brain region (node) as a power function, and the second by calculating a set of network statistics on two experimentally-derived network topologies. To assess rank-order connectivity, we compare both the linear and nonlinear networks in terms of a comparison of their coefficients and network statistics.

Network Structure Measures

Global indicators of network state are measured using network diameter and average clustering coefficient. The topological complexity of network structure is evaluated using the R^2 value of an exponential distribution.

Network diameter (*m*) is based on the *d* parameter of [57], and is calculated in Gephi 0.9.1 (Gephi consortium, Paris, France). This is formally defined as

$$m = \max_{v \in V} \epsilon(v) \quad \textbf{(2)}$$

where $\epsilon(v)$ is the maximum eccentricity of the graph, or the greatest distance between v and any other vertex in the network. This is a measure of the maximum path length between nodes within a network, and therefore represents the greatest distance information must travel from one node to reach another.

Parameter $\bar{C}$ is the average clustering coefficient [58], which is a measure of how densely nodes within the network are connected with neighboring nodes. This is calculated using Gephi 0.9.1 (Gephi consortium, Paris, France), and defined mathematically as

$$\bar{C} = \frac{1}{N} \sum_{i=0}^{N} C(i) \quad \textbf{(3)}$$

where N is the number of nodes in the network and C(i) is the local clustering coefficient. The local clustering coefficient measures how close an individual node is to being part of a fully-connected subnetwork.

R^2 represents a least-square approximation of an exponential distribution, and can be defined as

$$y = xB^{-\alpha} \quad \textbf{(4)}$$

where B is a constant greater than 1, $-\alpha$ is an exponent that defines the shape of the curve.
